# Renal Inhibition of Heme Oxygenase-1 Increases Blood Pressure in Angiotensin II-Dependent Hypertension

**DOI:** 10.1155/2012/497213

**Published:** 2011-11-16

**Authors:** Eva Csongradi, Megan V. Storm, David E. Stec

**Affiliations:** ^1^Department of Physiology and Biophysics, Center for Excellence in Cardiovascular-Renal Research, University of Mississippi Medical Center, 2500 North State Street, Jackson, MS 39216, USA; ^2^1st Department of Internal Medicine, Medical and Health Sciences Center, University of Debrecen, 4032 Debrecen, Hungary

## Abstract

The goal of this study was to test the hypothesis that renal medullary heme oxygenase (HO) acts as a buffer against Ang-II dependent hypertension. To test this hypothesis, renal medullary HO activity was blocked using QC-13, an imidazole-dioxolane HO-1 inhibitor, or SnMP, a classical porphyrin based HO inhibitor. HO inhibitors were infused via IRMI catheters throughout the study starting 3 days prior to implantation of an osmotic minipump which delivered Ang II or saline vehicle. MAP was increased by Ang II infusion and further increased by IRMI infusion of QC-13 or SnMP. MAP averaged 113 ± 3, 120 ± 7, 141 ± 2, 153 ± 2, and 154 ± 3 mmHg in vehicle, vehicle + IRMI QC-13, Ang II, Ang II + IRMI QC-13, and Ang II + IRMI SnMP treated mice, respectively (*n* = 6). Inhibition of renal medullary HO activity with QC-13 in Ang II infused mice was also associated with a significant increase in superoxide production as well as significant decreases in antioxidant enzymes catalase and MnSOD. These results demonstrate that renal inhibition of HO exacerbates Ang II dependent hypertension through a mechanism which is associated with increases in superoxide production and decreases in antioxidant enzymes.

## 1. Introduction

Heme oxygenases (HO) are the critical enzymes responsible for the breakdown of endogenous heme to biliverdin, carbon monoxide (CO), and free iron. Biliverdin is subsequently reduced to bilirubin and free iron is sequestered in the cell via induction of ferritin [1]. The metabolites of HO have a multitude of actions in the cardiovascular system. Two major isoforms of HO exist, the inducible form, HO-1, and the constitutively expressed isoform HO-2.

Several studies have demonstrated that systemic induction of HO-1 can prevent the development of hypertension in both experimental and genetic models of hypertension [2–5]. One study has even reported that HO-1 induction for 3 weeks resulted in a chronic 9-month lowering of blood pressure long after the levels of HO-1 had returned to normal [6]. Despite the experimental evidence for the antihypertensive effect of systemic HO-1 induction, the mechanism by which HO-1 induction lowers blood pressure is not known. One reason for the dependence of systemic induction of HO-1 is the lack of appropriate models which allow for organ or cell-type specific induction of HO-1.

Given the importance of the kidneys in the long-term control of blood pressure, renal induction of HO-1 could play a significant role in the antihypertensive effect of systemic HO-1 inducers [7]. Previous studies have demonstrated an important role for HO enzymes and their metabolites in the regulation of renal blood flow [8]. Moreover, intrarenal medullary interstitial (IRMI) infusion of a metalloporphyrin-based HO inhibitor, chromium mesoporphyrin (CrMP), attenuated renal pressure-natriuresis and resulted in the development of salt-sensitive hypertension [9]. We recently reported that kidney-specific induction of HO-1 via direct intrarenal medullary interstitial infusion of cobalt protoporphyrin (CoPP) attenuated the development of Ang II-dependent hypertension in the mouse [10]. The results of these studies highlight the importance of intrarenal HO in the regulation of blood pressure.

Traditional HO inhibitors are built upon metalloporphyrins which contain central metal atoms and serve as competitive inhibitors of HO enzymes [11, 12]. While these compounds are effective HO inhibitors, they also result in significant induction of HO-1 *in vivo* due to the metals that are utilized [13, 14]. Recently, a new class of imidazole-dioxolane HO inhibitors was described [15, 16]. These inhibitors are molecules which are similar in structure to heme, the natural HO substrate, but they do not contain metals and do not induce HO-1 when used *in vitro* [17]. We have previously demonstrated that the imidazole-dioxolane HO-1 inhibitor, QC-13, can effectively inhibit HO activity when administered *in vivo* either by *intraperitoneal* injection or IRMI infusion [18].

The role of renal medullary HO in the regulation of blood pressure in Ang II-dependent hypertension is not known. Previous studies have demonstrated that HO-1 is induced in the rat but not mouse kidney by Ang II [10, 19–22]. The goal of the present study was to determine the specific role of renal medullary HO-1 in the development of Ang II-dependent hypertension in mouse model by IRMI infusion of either a classical metalloporphyrin-based HO inhibitor, stannous mesoporphyrin (SnMP), or the imidazole-dioxolane HO-1 inhibitor, QC-13.

## 2. Methods

### 2.1. Animals

Experiments were performed on 12- to 16-week-old male C57BL/6J mice obtained from Jackson Labs (Bar Harbor, ME). The mice were fed a standard diet containing 0.29% NaCl and were provided water ad libitum. All animal protocols were approved by the Institutional Animal Care and Use Committee at the University of Mississippi Medical Center and performed in accordance with the *Guide for the Care and Use of Laboratory Animals* of the National Institutes of Health. Studies were performed on 16–20-week old male C57BL/6J mice (Jackson Labs, Bar Harbor, ME). All studies were performed in accordance with the approval of the University of Mississippi Medical Center Institutional Animal Care and Use Committee (IACUC) and in line with NIH guidelines. All mice underwent unilateral nephrectomy of the right kidney to remove potential contributions of the noninfused kidney to the blood pressure response to experimental manipulations. After seven days, intramedullary interstitial catheters were implanted 1.5–2 mm into the left kidney as previously described [10, 18]. Saline was then infused through the catheter for a period of 3 days after which the infusion was switched to QC-13, (2*R*,4*R*)-2-[2-(4-chlorophenyl)ethyl]-2-[(1*H*-imidazol-1-yl)methyl]-4-methyl-1,3-dioxolane hydrochloride (25 *μ*M, in saline), or stannous mesoporphyrin (SnMP, 400 *μ*M, in saline) in some mice (*n* = 6/group). Infusions were continued throughout the entire experimental protocol. Two days after the switch to QC-13 or SnMP mice were implanted with osmotic minipumps delivering either vehicle (saline) or Ang II at a rate of 1 *μ*g/kg/min. Five days after implantation of the osmotic minipumps, carotid artery catheters were implanted into the mice as previously described [10, 22]. After a 2-day recovery period, blood pressures were measured in 3-hour periods over the next 3 days in conscious, freely moving mice. Blood pressure data were continuously collected by a PowerLab 8/sp polygraph (AD Instruments, Denver, Colorado) and acquired on a computer running Chart 4 software provided by the manufacturer. Mice were euthanized after the last blood pressure recording session and organs harvested.

### 2.2. Heme Oxygenase Assay

Heme oxygenase assays were performed on lysates prepared from the renal medulla. Medullary protein lysates were prepared previously described [10, 22]. Briefly, tissue was homogenized in 250 mM sucrose, 10 mM KPO_4_, 1 mM EDTA, and 0.1 mM PMSF (pH 7.7) in the presence of protease inhibitors (2 *μ*g/mL aprotinin, leupeptin, and pepstatin). The homogenate was then centrifuged at 3,000 g for 15 min at 4°C and the supernatant was collected. Protein concentration was measured using a Bio-Rad protein assay with BSA standards. Reactions were carried out in a 1.2 mls containing: 2 mM glucose-6-phosphate, 0.2 unites glucose-6-phosphate dehydrogenase, 0.8 mM NADPH, 20 *μ*M hemin, and 0.5 mg of lysates as previously described [23]. The reactions were incubated for 1 hour at 37°C in the dark. The formed bilirubin was extracted with chloroform, and the change in optical density (ΔOD) at 464–530 nm was measured using an extinction coefficient of 40 mM/cm for bilirubin. HO activity was expressed as picomoles of bilirubin formed per hour per milligram of protein.

### 2.3. Western Blots

Western blots for HO-1 protein were performed on lysates prepared from the renal medulla as described above. Samples of 50 *μ*g of protein were boiled in Laemmli sample buffer (Bio-Rad, Hercules, CA) for 5 min and electrophoresed on 7.5% SDS-polyacrylamide gels and blotted onto nitrocellulose membrane. Membranes were blocked with Odyssey blocking buffer (LI-COR, Lincoln, NE) for 2 hours at room temperature, then incubated with the following antibodies: mouse anti-HO-1 monoclonal antibody (StressGen, Vancouver, Canada 1 : 2000), rabbit anti-HO-2 polyclonal antibody (StressGen), extracellular (EC), copper-zinc (CuZN), and manganese (Mn) superoxide dismutase (StressGen, Vancouver, Canada, 1 : 1000), and mouse antimonoclonal antibody (Sigma, St. Louis, MO, 1 : 10,000). 

Primary antibodies were used in conjunction with a mouse or rabbit anti-*β*-actin antibody (Gentest, 1 : 5,000) and incubated overnight at 4°C. The membranes were then incubated with Alex 680 goat anti-mouse or anti-rabbit IgG (Molecular Probes) and IRDye 800 goat anti-mouse or anti-rabbit IgG (Rockland, Gilbertsville, PA) for 1 hour at room temperature. The membranes were then visualized using an Odyssey infrared imager (Li-COR, Lincoln, NE) which allows for the simultaneous detection of two proteins. Densitometry analysis was performed using Odyssey software (LI-COR, Lincoln, NE). Levels of each specific protein are expressed as the ratio to *β*-actin for each sample.

### 2.4. Measurement of Renal Medullary Superoxide

Superoxide production in the renal medulla was measured using the lucigenin technique as previously described [22]. Briefly, kidneys were removed and separated into renal cortex and medulla. The medulla was then homogenized (1 : 8 wt/vol) in RIPA buffer (PBS, 1% Nonidet P-40, 0.5% sodium deoxycholate, 0.1% SDS, and a protease inhibitor cocktail; Sigma Chemical). The samples were centrifuged at 12,000 *g* for 20 min at 4°C. The supernatant was incubated with lucigenin at a final concentration of 5 *μ*M and NADPH at a final concentration of 100 *μ*M. The samples were allowed to equilibrate for 3 min in the dark, and luminescence was measured every second for 5–15 min with a luminometer (Berthold, Oak Ridge, TN). Luminescence was recorded as relative light units (RLU) per min. An assay blank with no homogenate but containing lucigenin was subtracted from the reading before transformation of the data. The protein concentration was measured using a Bio-rad protein assay with BSA standards. The data are expressed as RLU per min per milligram protein.

### 2.5. Statistics

Mean values ± SE are presented. Significant differences between mean values were determined by ANOVA followed by a post hoc test (Dunnett's). A *P* < 0.05 was considered to be significant.

## 3. Results

### 3.1. IRMI Infusion of HO Inhibitors Exacerbates Ang II-Dependent Hypertension and Increases Cardiac Hypertrophy

**Figure 1:**
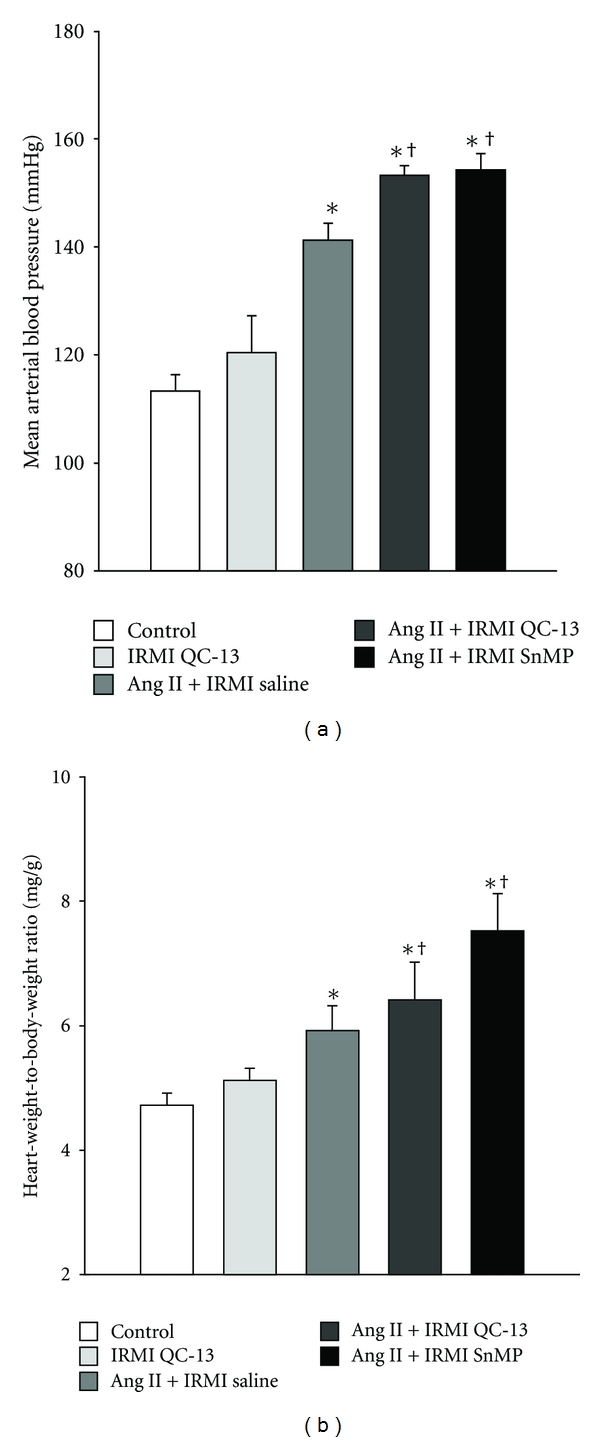
Mean arterial blood pressure (a) and cardiac mass index (b) in control, IRMI QC-13, Ang II + IRMI saline, Ang II + IRMI QC-13, and Ang II + IRMI SnMP-treated mice, *n* = 6/group. *P < 0.05 as compared to value in control mice. ^†^ *P* < 0.05 as compared to Ang II-treated mice. IRMI, intrarenal medullary interstitial infusion.

Mean arterial pressure (MAP) averaged 113 ± 3 mmHg in control mice and was slightly higher (120 ± 7 mmHg) in mice which received IRMI infusion of QC-13 alone. MAP was significantly increased to 141 ± 2 mmHg in Ang II-treated mice which received IRMI infusion of saline (Figure 1(a)). MAP was significantly augmented in Ang II-treated mice which received IRMI infusion of either QC-13 or SnMP and averaged 153 ± 2 and 154 ± 3 mmHg in each group, respectively (Figure 1(a)). Cardiac hypertrophy as indexed by the ratio of the heart weight to body weight was significantly increased in mice infused with Ang II as compared to control and IRMI QC-13-infused mice averaging 5.8 ± 0.3, versus 4.8 ± 0.2, versus 5 ± 0.2 mg/g in each group, respectively (Figure 1(b)). IRMI infusion of QC-13 or SnMP in Ang II treated mice resulted in further increases in cardiac hypertrophy as compared to Ang II treated mice alone with heart weight to body weight ratios averaging 6.4 ± 0.6 and 7.6 ± 0.6 mg/g in each group, respectively (Figure 1(b)). No differences in body weights were apparent between the groups with body weights averaging 26 ± 0.7, versus 27 ± 0.7, versus 25 ± 0.4, versus 25 ± 0.9, versus 25 ± 0.8 grams in control, QC-13, Ang II, Ang II + QC-13, and Ang II + SnMP-treated mice, respectively.

### 3.2. HO Activity and Protein Levels in the Medulla of Mice Receiving IRMI Infusion of QC-13 and SnMP

**Figure 2:**
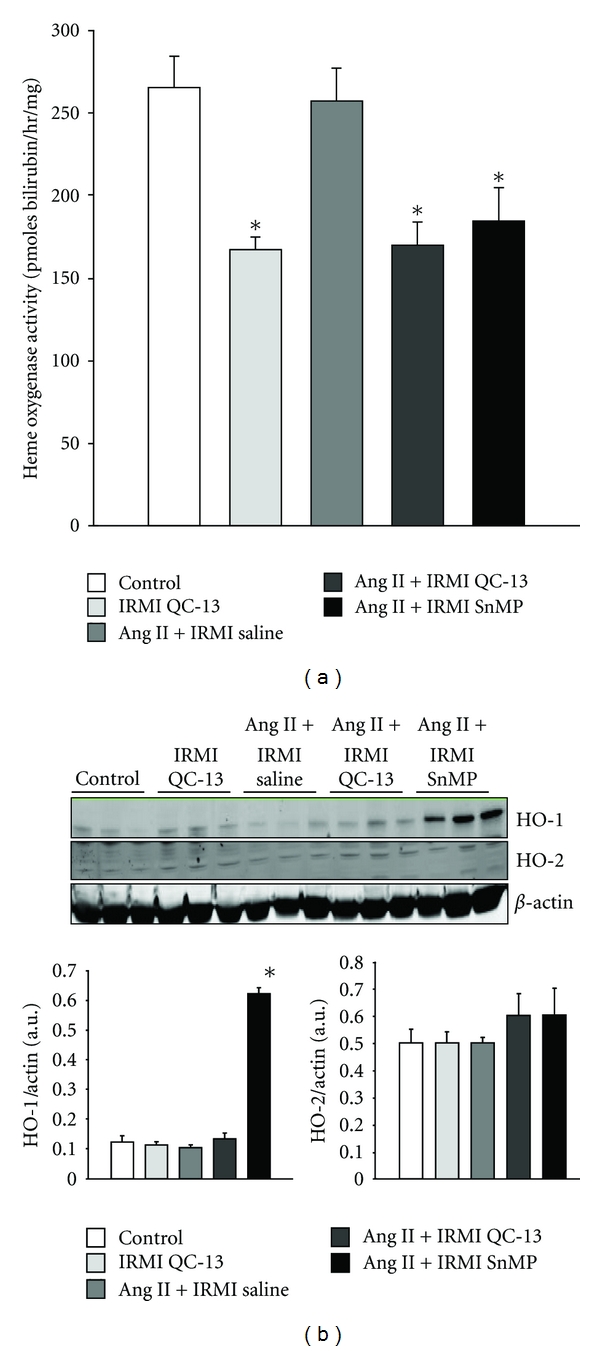
(a) Renal medullary heme oxygenase activity in control, IRMI QC-13, Ang II + IRMI saline, Ang II + IRMI QC-13, and Ang II + IRMI SnMP-treated mice, *n* = 6/group. (b) Representative western blots of HO-1 and HO-2 proteins from the renal medulla of control, IRMI QC-13, Ang II + vehicle, Ang II + IRMI QC-13, and Ang II + IRMI SnMP-treated mice, *n* = 3/group. *P < 0.05 as compared to value in control mice.

The effectiveness of IRMI infusion of the HO inhibitors, QC-13 and SnMP, to inhibit renal medullary HO was determined in each of the experimental groups. In agreement with our previous studies, HO activity was not affected by Ang II infusion (Figure 2(a)) [10, 22]. However, IRMI infusion of QC-13 either alone or in combination with Ang II treatment resulted in a 38% decrease in medullary HO activity as compared to levels in both control and Ang II-treated mice (Figure 2(a)). Likewise, IRMI infusion of SnMP also resulted in a similar level of HO inhibition in the renal medulla (Figure 2(a)). IRMI infusion of QC-13 had no effect on the levels of HO-1 protein in the medulla when administered alone or in combination with Ang II (Figure 2(b)). However, IRMI infusion of SnMP in Ang II-treated mice resulted in a significant induction of HO-1 in the renal medulla (Figure 2(b)). In agreement with previous results, Ang II infusion had no effect on the levels of HO-1 protein in the renal medulla (Figure 2(b)) [10, 22]. None of the different treatments had any significant effect on the levels of HO-2 protein in the medulla (Figure 2(b)).

### 3.3. Inhibition of HO Activity Increases Superoxide Production in the Renal Medulla

**Figure 3:**
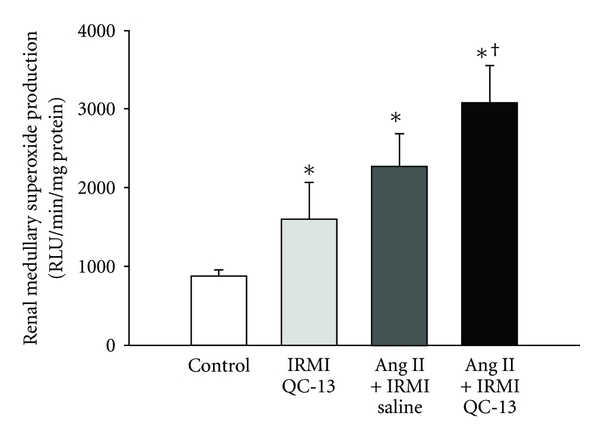
Renal medullary superoxide production in control, IRMI QC-13, Ang II + IRMI saline, and Ang II + IRMI QC-13-treated mice, *n* = 6/group. Renal medullary superoxide production was measured using the lucigenin technique as described in Section 2. *P < 0.05 as compared to value in control mice. ^†^ *P* < 0.05 as compared to Ang II-treated mice.

We determined the effect of renal HO inhibition with QC-13 on superoxide production in the renal medulla of control and Ang II-infused mice. Superoxide production was significantly increased by more than 2.5 times in the medulla of Ang II-treated mice as compared to control mice (Figure 3). IRMI infusion of QC-13 alone resulted in a significant increase in superoxide production in the medulla as compared to control mice (Figure 3). IRMI infusion of QC-13 in mice treated with Ang II resulted in an even greater increase in renal medullary superoxide production with levels 3.5-fold greater than those observed in control mice and 35% greater than those observed in Ang II-treated mice (Figure 3).

### 3.4. Inhibition of HO Activity and Antioxidant Proteins in the Renal Medulla

**Figure 4:**
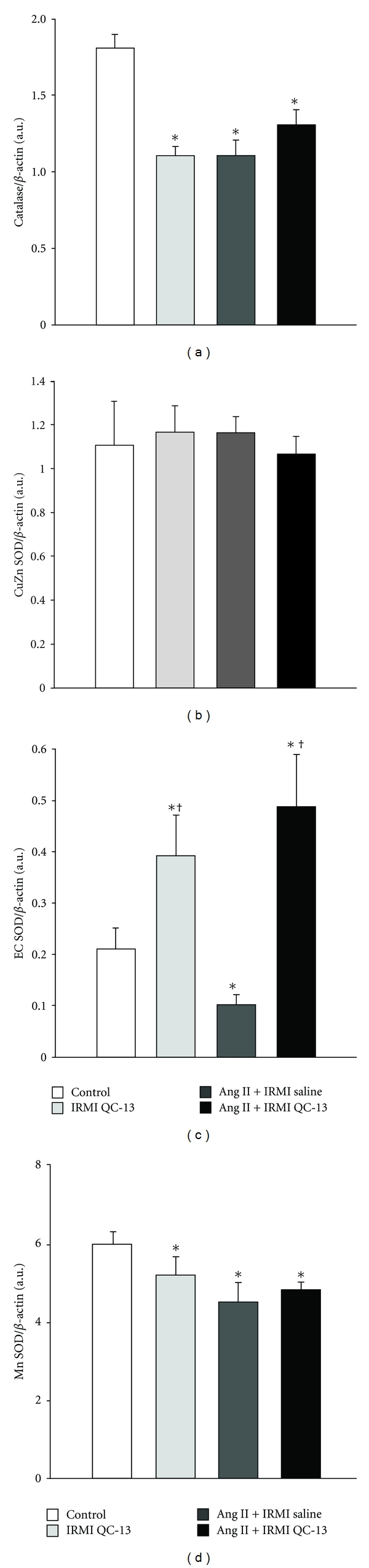
Representative western blots of catalase (a), copper-zinc (CuZn) superoxide dismutase (SOD) (b), extracellular (EC) SOD (c), and manganese (Mn) SOD (d) from the renal medulla of control, IRMI QC-13, Ang II + IRMI saline, and Ang II + IRMI QC-13-treated mice, *n* = 6/group. *P < 0.05 as compared to value in control mice. ^†^ *P* < 0.05 as compared to Ang II-treated mice.

We determined the effect of renal HO inhibition with QC-13 on the levels of antioxidant proteins catalase, CuZn, EC, and Mn SOD in control and Ang II-infused mice. IRMI infusion of QC-13 resulted in a significant decrease in renal medullary catalase protein which was equivalent to the decrease observed in Ang II-treated mice (Figure 4(a)). However, inhibition of HO activity during Ang II infusion did not result in further decreases in renal medullary catalase protein levels (Figure 4(a)). No differences in renal medullary CuZn SOD protein levels were observed between any of the experimental groups (Figure 4(b)). Ang II infusion resulted in a significant decrease in EC SOD levels as compared to control mice (Figure 4(c)). Interestingly, IRMI infusion of QC-13 either alone or in combination with Ang II treatment resulted in a significant increase in the levels of renal medullary EC SOD protein (Figure 4(c)). IRMI infusion of QC-13 resulted in a significant decrease in renal medullary Mn SOD protein which was equivalent to the decrease observed in Ang II-treated mice (Figure 4(d)). Inhibition of HO activity during Ang II infusion did not result in further decreases in renal medullary Mn SOD protein levels (Figure 4(d)).

## 4. Discussion

The present study was designed to determine the importance of renal medullary HO in the progression of Ang II-dependent hypertension. Renal medullary HO activity was inhibited by IRMI infusion of two separate HO inhibitors, QC-13, an imidazole-dioxolane HO inhibitor or SnMP, a classical metalloporphyrin-based HO inhibitor. IRMI infusion of either QC-13 or SnMP resulted in significant inhibition of HO activity and enhancement of Ang II-dependent hypertension as compared to mice infused with vehicle. Our present results confirm our previous observations that infusion of Ang II does not induce either HO-1 protein or increase HO activity in the mouse kidney [10, 22]. These results are opposite to the effect that Ang II infusion has on HO protein and activity in the rat kidney where several studies have demonstrated that Ang II treatment is an inducer of renal HO-1 [19–21]. The differences in the renal response to Ang II between the rat and mouse are not known and could be due to species differences in the promoters of the HO-1 gene or in the renal response to Ang II infusion.

Our finding that inhibition of HO activity in Ang II-infused mice exacerbates the blood pressure response and increases cardiac hypertrophy despite the lack of effect of Ang II on HO activity and protein in the kidney being similar to that recently described in a model of low Ang II infusion in the rat [24]. In this study, inhibition of HO activity resulted in increased renal vascular resistance and decreased renal blood flow in Ang II-infused rats despite no measureable changes in HO activity or protein in the kidney with the dose of Ang II-infused in the study. Similar effects on renal vascular resistance and blood flow were not observed in control rats in which HO was blocked. Interestingly, inhibition of HO activity resulted in a lowering of blood pressure in the Ang II-infused rats in this study as measured acutely under anesthesia [24]. While the differences in blood pressure responses between the studies could be due to species differences or differences in the way in which blood pressures were measured, the results from these two studies lend support for the hypothesis that HO-1 plays an important role in the stress response of the kidney even in conditions in which its activity is not significantly increased. One limitation in the current study is the lack of peripheral measurement of HO activity in the mice receiving IRMI QC-13 or SnMP. Thus, it is possible that systemic spillover of the inhibitors could have contributed to the increase in blood pressure in the mice receiving IRMI infusions of QC-13 or SnMP. However, in a previous study by our group, we could not detected induction of HO-1 in organs such as the liver and heart following IRMI infusion of CoPP [10].

The effect of renal medullary inhibition of HO in the present study is consistent with our previous results which demonstrated that renal-specific induction of HO-1 can prevent the development of Ang II-dependent hypertension [10]. They are also in agreement with previous studies in the rat demonstrating that renal medullary HO inhibition results in salt-sensitive hypertension [9]. One potential mechanism for the increase in blood pressure in mice treated with QC-13 is the effects of HO inhibition on regional renal blood flow. HO and its metabolites play an important role in the regulation of renal medullary blood flow which is important in the pressure-natriuretic response [8, 25, 26]. However, the effect of HO inhibition on renal hemodynamics in Ang II hypertensive mice has yet to be examined.

The current results demonstrate that inhibition of renal medullary HO activity was associated with an increase in superoxide production in the renal medulla. This observation is consistent with our previous study in which induction of HO-1 in the renal medulla was associated with a decrease in superoxide production [10]. Moreover, specific adenoviral overexpression of HO-1 in TALH decreased Ang II-dependent oxidative damage [27]. The link between HO-1 and its metabolites has also been strengthened in additional studies from cultured TALH cells which have demonstrated that increases in HO-1 protein, bilirubin, or CO can attenuate the development of Ang II-dependent superoxide production [28]. Superoxide anion is a major regulator of sodium reabsorption in the TALH through direct actions and through interaction with NO [29–31]. Previous studies in cultured mouse TALH cells have demonstrated that blockade of bilirubin formation by inhibition of the conversion of biliverdin to bilirubin resulted in increased Ang II-mediated superoxide production and enhancement of sodium transport [32]. Thus, it is possible that the increase in renal medullary superoxide production observed after HO inhibition results in increased sodium reabsorption in the TALH and enhancement of Ang II hypertension.

Intramedullary interstitial infusion of QC-13 alone resulted in a significant increase in superoxide production; yet it only resulted in a slight but not statistically significant increase in blood pressure. It is not clear why blood pressure did not increase further in the mice receiving QC-13 alone but it is possible that in the absence of a physiological stressor such as Ang II or high salt that increases in superoxide production are not able to increase blood pressure.

Inhibition of renal medullary HO activity with QC-13 resulted in significant alterations in important antioxidant enzymes control mice. In agreement with our previous studies, Ang II infusion also resulted in a significant decrease in renal medullary catalase levels which was not affected by blockade of HO-1 with QC-13 [10, 22]. Ang II infusion also resulted in a significant decrease in renal medullary Mn SOD levels which again were not altered by intramedullary inhibition of HO-1 with QC-13. The decrease in renal medullary catalase and Mn SOD observed in the medulla of mice infused with QC-13 alone suggests an important role for HO and its metabolites in regulating antioxidant enzymes in the kidney. Interestingly, inhibition of HO activity with QC-13 resulted in a significant increase in the levels of EC-SOD which could be a compensatory response to the increase in superoxide production observed in mice in which QC-13 was infused.

QC-13 is an imidazole-dioxolane HO inhibitor that has been reported to selectively inhibit the HO-1 versus HO-2 isoform [15, 17]. In agreement with our previous *in vivo* study, we did not detect any measureable induction of HO-1 in the renal medulla of mice in which QC-13 was administered via intrarenal medullary interstitial infusion [18]. This was in contrast to infusion of the classic porphyrin-based HO inhibitor, SnMP, which resulted in significant induction of HO-1 in the renal medulla. While a difference in the induction of HO-1 by these inhibitors was observed, they both resulted in similar degree of *in vitro *HO inhibition and an increase in blood pressure and cardiac hypertrophy in Ang II-infused mice. However, QC-13 has several technical advantages over porphyrin-based HO inhibitors like SnMP including water solubility and light insensitivity which make its use more appealing for chronic *in vivo* studies.

In summary, our results demonstrate that inhibition of renal medullary HO activity exacerbates Ang II-dependent hypertension and cardiac hypertrophy. The increase in blood pressure following HO inhibition in Ang II hypertension is also associated with an increase in renal medullary superoxide production and a decrease in antioxidant enzymes in the renal medulla. These results would suggest that renal HO-1 plays an important basal role to guard against further increases in blood pressure in Ang II hypertension through its antioxidant effects even in states in which it is not induced. Potential systemic effects of inhibitor spillover could also play a contribution to the increases in blood pressure. Further studies are needed to determine the precise roles of vascular versus tubular effects of HO inhibition as well as to determine the relative contributions of CO and bilirubin generation.
